# Evaluation of needle stick injuries among nurses of Khanevadeh Hospital in Tehran

**Published:** 2010

**Authors:** Mohammad Hassan Kazemi Galougahi

**Affiliations:** * Hygiene and Preventive Medicine Management, Health Office of Iranian Army (NEZAJA), Tehran, Iran Email: farshad.kazemi@yahoo.com

**Keywords:** Needle-stick injury, health personnel, needles

## Abstract

**BACKGROUND:**

Accidental needle-stick injuries (NSIs) are a hazard for health-care workers and general public health. Nursing workers are at high risk for occupational exposure to blood-borne pathogens (such as HBV, HCV and HIV) via sharp injuries of needle stick.

**METHODS:**

This descriptive analytical cross-sectional study was done on 158 nursing workers of Khanevadeh Hospital in Tehran to assess needle stick injuries prevalence and related factors via a questionnaire in 2008. Data were processed through SPSS _16.0_software using Pearson’s correlation coefficient, chi-square, independent t, and Fisher exact tests.

**RESULTS:**

About 40.5% of all participants were men and 59.5% were women. Mean age was 33.26 (8.03) years; 56.96% of participants had history of at least one needle stick injury and 22.15% of them had needle stick injury during last year. Injections were the most common action resulted to exposure (24.44%) and recapping of needles was at the second order (21.11%). Operation room had the highest prevalence (18.9%) of needle stick injuries among all wards of hospital. Emergency ward and ICU were next orders (15.6%). Exposed people believed that the most important and basic reason for needle stick injuries was patients crowdedness and hospital chaos (37.8%). There was no relation between ages, gender, years of professional life, education level and needle stick injuries but men used latex gloves less than women and did recapping needles more than them.

**CONCLUSIONS:**

The needle-stick injuries in nursing workers of Khanevadeh hospital (Tehran) were significantly less than other similar studies in Iran.

Needle-stick injuries (NSIs) are defined as “a penetrating wound with an instrument that is potentially contaminated with another person’s body fluid”.^1^ The United States National Institute of Occupational Safety and Health (NIOSH), defines needle stick injuries as injuries caused by needles such as hypodermic needles, blood collection needles, intravenous (IV) stylets, and needles used to connect parts of IV delivery systems.^2^

Injection is a skin perforating procedure done with a syringe and needle to enter a substance for preventive, treatment, or recreational purposes and is the most commonly used medical procedure. It is estimated that 12 billion injections are administered each year worldwide.^3^ Previous Studies show that more than 50 percent of the injections in developing countries are unsafe being resulted in serious health risks.^4^ Unnecessary injections are common in transitional and developing countries. It was shown that the average number of injections per person is 3.7 per year in these countries.^5^ United States Center for Disease Control (CDC) reported that about 600,000 to 800,000 needle stick injuries occur annually among 8 million health care workers in the United States that work in hospitals and other health care settings.^6^

Accidental needle-stick injuries are a hazard for health-care workers and for the general public.^7^ Medical, dental, nursing, and midwifery workers are at high risk for occupational exposure to blood-borne pathogens (BBPs) via sharp injuries such as needle stick injuries.^8^ According to the World Health Organization, 16000 cases of hepatitis C (HCV), 66000 cases of hepatitis B (HBV), and 1000 cases of HIV might occur worldwide in the year 2000 among health care workers through their exposure to NSIs.^9^ The risk of transmission of infections from the patient to the healthcare worker by needle stick injuries in hepatitis C, hepatitis B and HIV is 3%, 30% and 0.3% respectively. Moreover, transmission risk depends on the viral load of the patient and the amount of blood passes from one to the other.^10^ Accidental NSIs result in more than 100000 injuries in health-care workers in UK hospitals annually.^11^ The major activities causing needle stick injuries are administering injections, blood sampling, recapping needles, needles disposal, handling trash and dirty linen (downstream injuries), and while transferring blood or any body fluid from a syringe to a specimen container (such as a vacuum tube) and misses the target.^2^ Safety practice has a serious role in safety and health maintenance of health care workers.^11^

As NSI is an important risk factor in nursing workers of medical centers, we decided to evaluate prevalence of NSI in nursing workers of Tehran Khanevadeh Hospital and identify related factors in order to decrease the risk of infectious diseases transmission.

## Methods

This descriptive analytical cross-sectional research was performed on a population composed of nursing workers of Tehran Khanevadeh Hospital to investigate the prevalence of needle stick injuries and some related effective factors in 2008. We used “all of available cases” sampling method and 158 nursing workers participated in this study. Inclusion criteria were having nursing certificates and related fields possession, working in Tehran Khanevadeh Hospital, and participant’s consent.

Data gathering tool was a researcher-made questionnaire including 17 questions (4 demographic and 13 NSI related questions) asked about bottom mentioned factors either in multiple choice form or open form: gender, age, years of professional life, education level, existence/not existence of safety box, recapping/unrecapping of needles, needle stick history, number and time of NSIs, the action resulting in NSIs, using/not using of latex gloves, contaminated/not contaminated of exposed needles with patients’ blood or discharges, ward of injury happening, predisposing reason of NSIs, state of hepatitis B vaccination, and post injury prophylaxis. Validity of questionnaire was confirmed by experts’ judgment and literature reviews. To evaluate questionnaire’s reliability, 10% of participants were retested again 2 weeks later. The reliability of questionnaire was confirmed by Cronbach’s alpha calculation (α = 0.87).

The questionnaire was submitted to all 158 nursing workers of this hospital to be filled in after a brief description by researcher. After filling up questionnaires, acquired data was processed through SPSS_16.0_ software. Data were analyzed by statistical tests including Pearson’s correlation coefficient, chi-square, independent t, and Fisher exact tests.

## Results

The mean age of studied population was 33.26 (8.03) years in which the youngest was 17 and the oldest was 55 years old. The average year(s) of working was 13.53 (7.96) years (with the minimum of 2 months, the maximum of 29 years). Mean number of NSIs occurrence in studied population was 3.99 (5.68). Of them all, 22.15% mentioned at least one exposure to NSIs in last year, 46.2% during last 5 years, and 56.96% during professional life. Participants’ characteristics are presented in table 1.

**Table 1:** Characteristics of studied nursing workers (n = 158)

Gender	Percent	n
Male	40.5	64
Female	59.5	94
Age group:		
< 20	2.5	4
20-29	35.4	56
30-39	36.7	58
40-49	22.2	35
≥50	3.2	5
Education level:		
High school diploma	37.3	59
College graduate	12.7	20
BS graduate	48.1	76
MS graduate	1.9	3
Year(s) of professional life:		
< 10	39.2	62
10-20	35.4	56
≥21	25.3	40
Recapping needles:		
Yes	44.3	70
No	55.7	88
Number of NSIs during professional life:		
0	43.04	68
1	24.05	38
2-5	23.42	37
6-10	5.7	9
≥11	3.8	6
Safety box existence:		
Yes	95.6	151
No	4.4	7

Injections were the most common actions resulted to NSIs (24.44% of all exposures). Operating room was the most prevalent site of NSI occurrence (18.9%) and other wards were emergency ward and ICU (15.6%), obstetrics and gynecology ward (10%), operation ward (6.7%), laboratory, orthopedic ward and delivery room (5.6%), recovery, general ward, children’s ward, internal ward, injection ward, lithotripsy ward, and infirmary (2.2%) and neurosurgery ward (1.1%) respectively.

Characteristics of NSIs exposed cases are shown in table 2.

**Table 2:** Characteristics of NSIs in exposed group (n = 90)

Type of action resulted to NSIs	%	n
Injections	24.44	22
Recapping needles	21.11	19
Angiocatheter appliance	18.89	17
Blood sampling	15.55	14
Suturing	11.11	10
Liquid extraction from vials	8.89	8
Using latex gloves at injury time:		
Yes	42.2	38
No	57.8	52
HBV vaccination at injury time:		
Complete (thrice) vaccination	41.1	65
Incomplete vaccination	10.8	17
No vaccination	5.1	8
Main predisposing factor of NSIs:		
Ward crowdedness and chaos	37.8	34
Imprudence	24.4	22
Colleague imprudence	14.4	13
Exhaustion	13.3	12
Inaccessibility to safety equipments	10	9
Device contamination by patients’ blood or discharges:		
Yes	72.2	65
No	27.8	25

After NSIs, 5.6% of nurses washed the wound by water and soap only, 5.7% squeezed the wound place too, 70% disinfected the wound by antiseptics too, and 14.4% after using antiseptics, tested patient’s blood for HIV or HBV in laboratory in order to be assured.

We tested relationship between gender and NSIs occurrence by chi-square test, but there was no significant relationship (p = 0.186).

There was a meaningful relationship between gender and using latex glove (p = 0.044) which means that women had used latex gloves more than men (using chi-square test).

We did not observe any meaningful relationship between gender and HBV vaccination (p = 0.073).

We found a relationship between gender and recapping needles (p = 0.013) using chi-square test meaning that men recapped needles more than women.

There was no relationship between age and NSIs occurrence (p = 0.125) and also between age and number of NSIs in exposed persons (p = 0.554) using Independent t-test and Pearson’s correlation coefficient.

We tested relationship between years of professional life and NSIs occurrence by independent t-test but we could not find any relationship between them (p = 0.24). We also could not found any relationship between years of professional life and number of NSIs in exposed persons using Pearson’s correlation coefficient (p = 0.680).

There was a relationship between age and recapping needles (p = 0.025) using independent t-test; Persons who did recapping were younger than others.

We observed a relationship between years of professional life and recapping needles (p = 0.019) using Independent t-test; Persons who did recapping needles had lesser work experience.

There was no relationship between education level and NSIs occurrence (p = 0.709) using Fisher’s exact test.

## Discussion

About 22.15% of nursing workers had at least one exposure to NSIs in last year, 46.2% during last 5 years, and 56.96% during professional life.

Lotfi and Gashtasbi studied 90 nurses, midwives, physicians, laboratory technicians, operation technicians, nurse aids, and anesthesia personnel in Astara and observed that 67% of personnel had at least one needle stick injury in the last year. Only 20% of them had not any injury from needle stick during entire work career.^12^ The prevalence of NSIs in this study was higher than our research.

Nasiri et al performed a research on 352 staff of educational and non-educational hospitals of Mazandaran province in 2003-2005. The results showed that 75.6% of staff experienced infective and blood borne pathogens from a patient that has infection at least once. The most common damage (72.6%) was due to syringes.^13^ The main source of NSIs in this study was similar to our research but prevalence of NSI was higher.

Aghadoost et al studied 678 students and staff of educational-medical centers of Kashan University of Medical Sciences. Ninety four percent of participants and 100% of emergency nurses, operation room technicians, and laboratory technicians reported at least one episode of blood exposure in their professional life. Rate of blood exposure via needle stick was 58.2%. The highest rate of blood exposure (31.6% of all injuring procedures) was related to injection and the most common device resulted to NSIs (37.5% of all injuring devices) was needle used for injections.^14^ The main source of NSIs in this study was similar to our research but prevalence of NSI was higher.

Mantel et al surveyed the staff of 120 health facilities of Syrian Arab Republic in 2007 and showed that 14% of the staff reported needle-stick injuries in the previous 12 months.^15^ The prevalence of NSIs in this study was less than our research.

In our study, 56.96% of participants had at least one exposure to NSIs during their professional life and 22.15% were exposed to NSIs during last year. The NSIs in nursing workers of this hospital were significantly less than similar studies in Iran. Injection was the most common activity led to injuries similar to previous researches.

## Conclusions

Of all the studied population, 44.3% recapped needles in spite of safety box existence in 95.6% of wards. So recapping process was 21.57% of all NSIs causes. This high rate shows that health workers systematic education is essential and plays an important role in infectious diseases prevention.

None or incomplete HBV vaccination in 27.8% of exposed participants was an important deficit. Because of high exposure probability in nursing workers to hepatitis B through patients’ blood, regular HBV vaccination and antibody control are important preventive actions.

Required educations about post-exposure prophylaxis and latex gloves usage during working hours are other administrative points for nursing workers.

These preventive actions should be performed in medical centers particularly in high risk wards such as operation room, emergency and ICU.

The author declares no conflict of interest in this study.
